# Joining of Polyethylene Using a Non-Conventional Friction Stir Welding Tool

**DOI:** 10.3390/ma15217639

**Published:** 2022-10-30

**Authors:** Miguel A. R. Pereira, Ivan Galvão, José Domingos Costa, Rui M. Leal, Ana M. Amaro

**Affiliations:** 1 Department of Mechanical Engineering, CEMMPRE, University of Coimbra, 3030-788 Coimbra, Portugal; 2 ISEL, Departamento de Engenharia Mecânica, Instituto Politécnico de Lisboa, Rua Conselheiro Emídio Navarro 1, 1959-007 Lisbon, Portugal; 3 LIDA-ESAD.CR, Instituto Politécnico de Leiria, Rua Isidoro Inácio Alves de Carvalho, 2500-321 Caldas da Rainha, Portugal

**Keywords:** friction stir welding, polyethylene, stationary shoulder, heated tool, joint efficiency, weld defects

## Abstract

The objective of the current study was to butt-weld 6 mm-thick polyethylene (PE) plates by friction stir welding (FSW) using a non-conventional stationary shoulder tool. The welds were performed with an unheated shoulder and with a shoulder temperature of 85 °C. Additionally, rotational speeds of 870, 1140 and 1500 rpm; welding speeds of 60 and 120 mm/min; and plunge depths of 5.5 and 5.7 mm were used. The influence of these parameters on morphology, hardness, ultimate tensile strength, elongation at break and fracture modes was evaluated. Shoulder heating proved to be crucial for the optimization of PE joints by FSW, as it clearly improved joint efficiency. Furthermore, shoulder heating promoted the reduction in internal and external defects, such as porosity and surface burning. Defect-free weld seams were obtained with higher rotational speeds and a lower welding speed. A maximum joint efficiency of about 97% was achieved with a shoulder temperature of 85 °C, a rotational speed of 1500 rpm, a welding speed of 60 mm/min and a plunge depth of 5.7 mm. A weld with the average joint efficiency of 92% was produced at 120 mm/min, which based on the literature found is the highest welding speed reported that achieved a joint efficiency above 90%.

## 1. Introduction

The current range of applications for polymers is very wide. The need to reduce vehicles’ weight, for example, requires the use of lighter materials with good mechanical properties, such as good stress-to-weight ratios and toughness [1,2]. In addition, the need to manufacture larger and more complex products often demands the division of components into two or more parts that later must be joined. Typically, polymeric materials are joined by means of adhesives, mechanical fastening or welding. Adhesive joining usually requires detailed joint preparation, surface cleaning and large cure periods. Furthermore, adhesives tend to degrade faster when exposed to aqueous environments [3]. On the other hand, mechanical fastening entails an increase in weight, due to the use of rivets or screws, in addition to the stress concentration in the holes required in these technologies. Lastly, mechanical fastening joints are currently not watertight [4,5]. Hot plate, hot gas, ultrasonic welding and laser welding are some of the welding methods currently used to join polymers, while friction stir welding (FSW) is a more recent application in polymeric materials [6].

FSW was developed and patented in the United Kingdom by The Welding Institute in 1991 [7]. The process was initially developed to overcome the difficulties of aluminum alloys-joining by conventional welding methods. Currently, FSW is also being used to join other metallic materials, such as copper, titanium, steel and magnesium [8]. Furthermore, the possibility of joining dissimilar metallic materials has been investigated [9,10]. 

The joining of polymeric materials by FSW was first demonstrated in 1997 [11,12]. Nonetheless, the technology is only suitable for recyclable polymers, i.e., thermoplastics, because they soften when exposed to high temperatures without suffering chemical transformation, while thermosets degrade due to irreversible molecular changes [13,14]. Among thermoplastics, polyethylene (PE) is one of the most popular, being widely used in industrial and commercial products [14].

The tools for FSW are generally composed of two parts, the shoulder, which generates most of the heat in the process, and the pin. In the conventional process, both rotate simultaneously [15]. Heat is generated by friction between the tool and the polymeric material, and by severe plastic deformation, resulting in the consequent softening of the polymer. Beyond that, the pin is mainly responsible for mixing the softened material of the interfaces to be joined, while the shoulder is responsible for avoiding material projection out of the weld seam [11,12]. FSW can be performed in various joint configurations, although the most studied are the butt and the lap joint configurations [16]. FSW in lap joint configuration is also known as friction stir lap welding (FSLW) [17]. FSW does not require any filler material, protective atmosphere, joint preparation or post-treatment. In addition, FSW does not generate toxic fumes or UV radiation emissions during welding [18,19,20]. The main welding parameters involved are rotational speed, welding speed, plunge depth, axial force, tilt angle, tool geometry and tool dimensions. For polymers, material temperature and tool temperature can be included as important parameters as well, as justified below.

Due to the poor thermal conductivity and low melting temperature of polymers, previous studies demonstrated that, typically, the conventional FSW tool is not suitable for welding polymers. The heat generated by a rotating shoulder is concentrated on the surface of the polymer, promoting material burning and heavy material projection, commonly known as flash defect [21,22]. Consequently, the tensile strength of welds produced by conventional FSW is normally reduced. For example, Mishra et al. [15] and Sheikh-Ahmad et al. [22] achieved maximum joint efficiencies of 44% and 66%, respectively, for the butt-welding of high-density polyethylene (HDPE). In both cases, all welds presented internal and external defects, such as internal voids and surface burr. Saeedy and Givi [23,24] butt-welded 6 mm- and 8 mm-thick medium-density polyethylene (MDPE) and obtained maximum joint efficiencies of 70% and 75%, respectively. Despite having significant visible flash defects, Mustapha et al. [25] achieved joint efficiencies of about 80% on HDPE butt welds. Nevertheless, Bozkurt [1] managed to butt-weld 4 mm-thick HDPE with joint efficiencies above 90% with conventional FSW tools.

Material preheating was one of the solutions investigated to improve the efficiency of polymer joints by FSW. Squeo et al. [26] performed preliminary studies on the subject and demonstrated their benefits for the butt-joining of 3 mm-thick HDPE. Aydin [4] achieved joint efficiencies of 89% for the butt-joining of 4 mm-thick preheated ultra-high molecular weight polyethylene (UHMWPE). However, since conventional welding tools were used, a poor surface finish was generally obtained in the aforementioned studies. Rehman et al. [27] and Sheikh-Ahmad et al. [28] managed to produce welds with an ultimate tensile strength that was greater than the yield strength of the parent material for the joining of 6 mm-thick HDPE with a conventional tool and a PTFE shoulder.

Another solution investigated was the use of heated tools. Different alternatives were evaluated regarding heating methods. For example, Vijendra and Sharma [29] investigated the potential of induction heated tools, which required the use of an induction system around a conventional welding tool. They performed bead-on-plate welds and obtained specimens with ultimate tensile strength above the yield strength of the parent material. Still, the true potential for joining polymeric materials needs further investigation.

One of the methods that proved to be the most successful to join polymers was the use of stationary shoulder tools. Stationary shoulder tools reduce the formation of surface defects on polymers [2], as demonstrated by Romero et al. [30] after comparing the performance of conventional and stationary shoulder tools for the butt-welding of 8.5 mm-thick HDPE. Still, the loss of heat produced by a rotating shoulder must be compensated in order to achieve sound welds, as stated by Eslami et al. [2].

In that regard, Nelson et al. [31] developed and patented the hot shoe tool. This type of tool is characterized by a long rectangular stationary shoulder, known as the shoe, that can be heated by an internal heating system. In addition to producing a good surface finish, as it combines all the benefits of a stationary shoulder, the addition of heat improves material mixing and reduces the formation of internal porosity by offering controlled cooling under pressure. The application of hot shoe was further investigated by other researchers, and the conclusions converged, proving that the different hot shoe tool designs easily outperform conventional tools, achieving greater joint quality and, therefore, stronger welds [32]. For example, Azarsa and Mostafapour [33], Mostafapour and Azarsa [34], and Azarsa et al. [12] butt-joined 10 mm-thick HDPE plates using hot shoe tools and managed to achieve up to 96% of base material flexural strength. 

With the idea of developing a more sustainable stationary shoulder tool that did not require the use of an internal heating system, Eslami et al. [35] developed a new type of PTFE stationary shoulder tool, which uses a highly heat conductive sleeve around the pin. By means of friction, the sleeve temperature increases, preheating the material before tool passage. With this solution, joint efficiencies greater than 95% were achieved for the butt welding of 3 mm-thick high-molecular-weight polyethylene (HMWPE) [2,21].

Finally, the removal of the root defect, a welding defect typically found on butt joints, demanded the investigation of new FSW methods. The root defect corresponds to the existence of an unwelded area at the bottom of the joints. The dimension of the root defect can be controlled by adjusting the length of the pin, but it is difficult to completely remove by conventional FSW. Nonetheless, the root defect can be successfully eliminated by performing a double side passage of the tool, as demonstrated by Arici and Sinmaz [5]. By applying this technique, Saeedy and Givi [36] achieved a maximum joint efficiency of 80% for the butt-welding of 8 mm-thick HDPE, and Arici and Selale [37] obtained a maximum joint efficiency of 87% for the butt-joining of 5 mm-thick MDPE. The double-pass technique is expensive due to the time consumed, so the single-pass technique should be optimized.

The aim of the present work was to butt-weld PE plates using a non-conventional stationary shoulder tool. Furthermore, the effect of shoe temperature, rotational speed, welding speed and plunge depth on the morphology, hardness, ultimate tensile strength, elongation at break and fracture mode was investigated.

## 2. Materials and Methods

### 2.1. Base Material Properties

White PE plates that were 6 mm-thick, 80 mm-wide and 220 mm-long were welded in a butt joint configuration. The main properties of the PE are listed in Table 1.

Regarding the ultimate tensile strength property, the experiments revealed that the plates used were slightly anisotropic. In the longitudinal direction, the ultimate tensile strength of the parent material was 24.2 MPa. In the transversal direction, the material revealed an ultimate tensile strength of about 25.8 MPa. The welding was always performed in the longitudinal direction. This means that the specimens were tested in the transverse direction. This information is particularly important as it interferes with the calculation of joint efficiency. The joint efficiency is the ratio between the mechanical strength of the joint and the strength of the parent material [32]. In this case, the strength of the base material considered was 25.8 MPa. 

### 2.2. Experimental Setup and Welding Tool

The plates were positioned on a clamping device to avoid separation during the welding process. The welding was conducted on a conventional milling machine. This machine allows the variation of plunge depth, rotational speed, welding speed and tilt angle. It operates in position control and does not measure axial force. 

**Figure 1:**
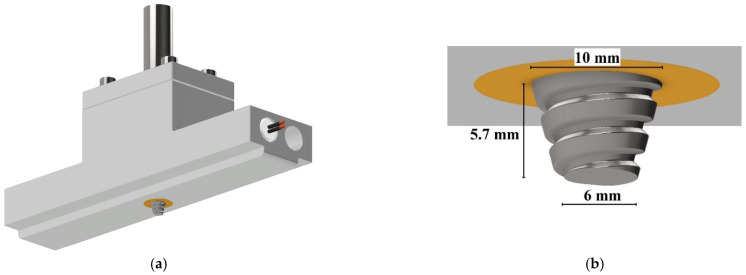
Schematic illustration of the tool used: (**a**) general view and (**b**) pin close-up view.

Welding was performed with a long rectangular stationary shoulder tool developed by Mendes et al. [39,40], which is an adaptation of the hot shoe tool design. The tool used is schematically illustrated in Figure 1a. Unlike most hot shoe tools, the pin is located in the center of the shoulder and not on an edge. In addition to allowing cooling under pressure at a controlled temperature after pin action like in most hot shoe tools, this tool allows the preheating of the base material before pin action. To evaluate the effect of shoulder temperature during welding, the tool was designed with two holes at each end, allowing the use of a maximum of 4 cartridge heaters. Preliminary experiments determined that two 400 W cartridge heaters, one on each side and diagonally positioned, were sufficient to evenly heat the shoulder to the desired temperature. The shoulder temperature was measured with a J-type thermocouple, and an on–off control system was implemented. The stationary shoulder is made of 5083 aluminum alloy and has a contact surface with a polymer of 180 mm × 25 mm. The pin is made of H13 hardened steel, quenched and tempered to a hardness of 50 HRC; has a conical threaded geometry, a length of 5.7 mm, a base diameter of 10 mm and a tip diameter of 6 mm, as illustrated in Figure 1b. The threads have 2 mm of pitch distance and a metric profile. Since the pin was produced with left-handed threads, the machine was operated in the clockwise direction in order to promote a better descendent vertical material flow of the plasticized polymer, as suggested by [41]. 

### 2.3. Welding Parameters

Plunge depths of 5.5 and 5.7 mm; rotational speeds of 870, 1140 and 1500 rpm; and welding speeds of 60 and 120 mm/min and a zero-tilt angle were used in this study. In addition, the shoulder temperature was evaluated, and two conditions were compared. Welds were performed without shoe heating and with the shoe at 85 ± 5 °C. The varying parameters, their levels and values are shown in Table 2.

Twelve welds were produced under different welding conditions. The protocol of the welding tests is represented in Table 3. The designation of each experiment summarizes the different welding conditions used. Welds produced with and without heated shoe are designated by the prefix PEH or PE, respectively, followed by the rotational speed and welding speed used. 

Instead of using the Taguchi method to define the design of experiments, as in most studies, a parameter-by-parameter approach was used. The Taguchi method is an important tool used to find optimum values while studying a significant number of parameters with fewer experiments [1]. However, a more conventional approach was chosen, as it facilitates the comparison between experiments, permitting a better understanding of the influence of each parameter. 

### 2.4. Characterization Methods

The morphology of the welds was evaluated by visual inspection and by using a Leica DM400M LED optical microscope. Tomography analyses were carried out using a Bruker Skyscan X-ray microtopographer and were used to improve the characterization of the internal morphology of the welds. Vickers microhardness measurements were performed on an HMV-G Shimadzu tester, with a testing load of 200 g and a dwell time of 15 s. A total of 51 indentation points were evaluated along 25 mm in each weld, resulting in a spacing between indentations of 0.5 mm. The measurements were carried out at half the thickness of the specimens, taking care to coincide the point 25 with the center of the weld. This procedure allowed the evaluation of the hardness of all zones, i.e., the base material (BM) and heat-affected zone (HAZ) of the advancing side (AS), the stir zone (SZ), and the HAZ and BM of the retreating side (RS). The tensile strength was evaluated using a Shimadzu AGS-X 100kN universal testing machine, with a testing speed of 5 mm/min. Specimens were prepared and tested in accordance with the standard ASTM D638-14. Local strain fields were acquired by digital image correlation (DIC) using the Aramis 3D 5M optical system (GOM GmbH). The precise value of the elongation at break of the PE was not determined, as the base material specimens did not break with the displacement limit of the tester. It was only possible to determine that the strain at break of the PE is greater than 500 mm, which corresponds to an elongation of more than 850%. 

## 3. Results and Discussions

### 3.1. Morphological Analysis

**Figure 2:**
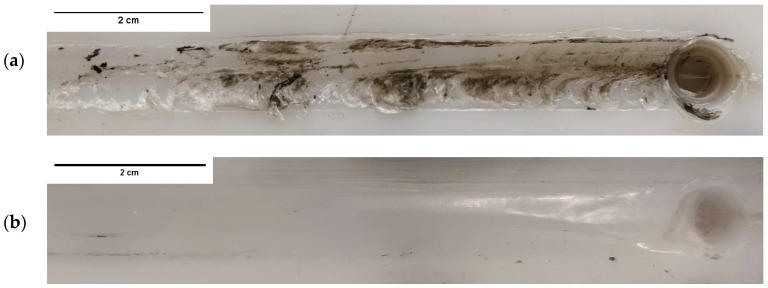
Surface finish of welds (**a**) PE_1500_60 and (**b**) PEH_1500_60.

A visual inspection of the weld surfaces shows that rotational speed, welding speed and shoe temperature greatly influenced the surface finish of the welds. Generally, welds produced with unheated shoe presented large, burned areas and an irregular surface finish. Due to the increase in heat generated by friction, these defects worsened by increasing the rotational speed and decreasing the welding speed. For these reasons, the worst surface finish was obtained in the weld PE_1500_60, as shown in Figure 2a. Although it may seem counterintuitive, when external heat was added, burning defects did not occur. Welds produced with heated shoe presented defect-free smooth surfaces, without visible degraded material. Even at the highest rotational speed and the lowest welding speed, when the shoe was heated at 85 °C the resulting weld surface was completely free of defects, as illustrated in Figure 2b. The removal of material degradation by heating the stationary shoe was previously reported by Mostafapour and Azarsa [34] and Azarsa et al. [12]. 

The literature suggests that PTFE shoulders or PTFE coatings can be used to improve the quality of the welds, as they avoid material adhesion to the tool, therefore, contributing to the elimination of surface defects [12,27,34]. Furthermore, Rezgui et al. [42] observed that stationary shoulders made of low thermal conductivity materials, such as PTFE, improve heat distribution by retarding its colling rate. Still, when external heat was added in the current study, the uncoated AA 5083 hot shoe achieved excellent results, producing smooth and homogeneous weld surfaces that were free of burns or other types of welding defects. The resulting welds were particularly difficult to distinguish from the surrounding base material, as shown in Figure 2b. Still, all welds presented residual depressions along the weld surfaces due to material shrinkage during cooling, a typical phenomenon on FSW of polymers. Similar observations were reported by Sheikh-Ahmad et al. [22].

**Figure 3:**
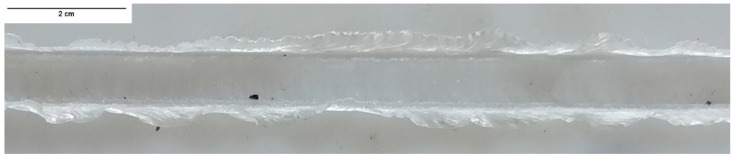
Flash defect on the surface of the weld PE_1140_60.

Although most welds were produced with a plunge depth of 5.7 mm, the weld PE_1140_60 was produced with a penetration of only 5.5 mm, resulting in a gap of 0.2 mm between material surface and shoulder bottom surface. This gap allowed material to escape from the weld seam, leading to the formation of a significant amount of burr. For this reason, a heavy flash defect was formed, as shown in Figure 3. Flash defects were not detected in any other weld. 

**Figure 4:**
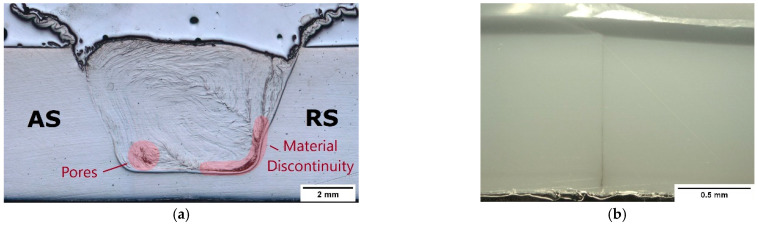
Microscope images of the cross-section of weld PE_1140_60. (**a**) General view and (**b**) root defect close-up view.

Additionally, the cross-section macrograph of this weld, shown in Figure 4a, evidence that the reduction in plunge depth contributed to the formation of voids, such as internal porosity and material discontinuity, especially along the border of the SZ on the RS. The formation of these defects was expected, since there was less polymer in the interior of the weld seam due to material escaping. Although all welds presented root defect, the reduction in penetration further contributed to the formation of the largest root defect (1.15 mm) on weld PE_1140_60. As it was difficult to perceive this defect in Figure 4a, a close-up view of the root defect is shown in Figure 4b.

**Figure 5:**
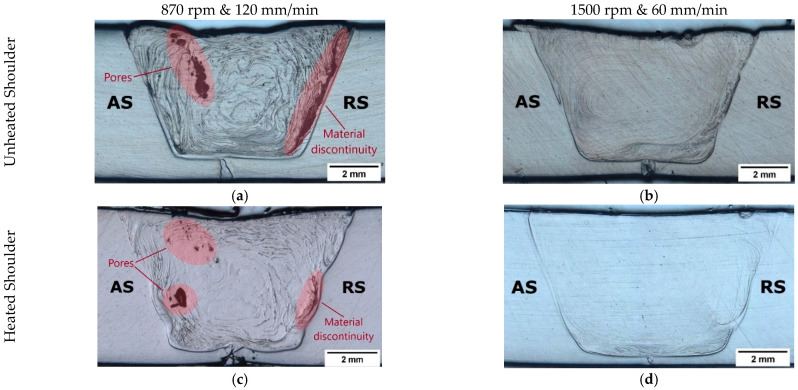
Microscope images of the cross-section of welds (**a**) PE_870_120, (**b**) PE_1500_60, (**c**) PEH_870_120 and (**d**) PEH_1500_60.

Although plunge depth is the main parameter influencing the root defect, other parameters indirectly affect it as well. The increase in rotational speed and the reduction in welding speed increased the heat generated by friction. Consequently, larger SZs were formed, favoring the reduction in root defects, as reported by other authors [12,24,27,34]. Shoe heating further increased the SZ growth, minimizing root defects. The macrographs of the cross-sections of welds PE_870_120, PE_1500_60, PEH_870_120 and PEH_1500_60 are shown in Figure 5. Weld PE_870_120, Figure 5a, presented the smallest SZ (38 mm^2^) and the largest root defect (900 µm) among the welds produced with a plunge depth of 5.7 mm. By increasing the rotational speed and decreasing the welding speed, weld PE_1500_60, Figure 5b, showed an increase in SZ area (40 mm^2^) and a reduction in root defect (650 µm). A similar effect was observed when using shoe heating. In comparison to weld PE_870_120, weld PEH_870_120, Figure 5c, revealed a slightly larger SZ (38.5 mm^2^) and a shorter root defect (500 µm). Weld PEH_1500_60, Figure 5d, presented the largest SZ (47 mm^2^) and the smallest root defect (350 µm). Similar observations were reported by Rehman et al. [27] and Sheikh-Ahmad et al. [28]. 

From Figure 5, several other conclusions regarding the influence of the different parameters on material flow and internal porosity can be drawn. For example, the onion ring flow lines of the SZ are very useful for a better understanding of the type of material flow of each weld. The onion rings are special types of flow lines that close on themselves, forming concentric lines [43]. The flow lines of welds PE_870_120, Figure 5a, and PEH_870_120, Figure 5c, indicate that turbulent material flow occurred during welding, as they are irregular and very pronounced. On the other hand, the flow lines of welds PE_1500_60, Figure 5b, and PEH_1500_60, Figure 5d, are straighter, smoother and in higher number, proving that the material flow was improved by increasing the rotational speed and decreasing the welding speed. The higher heat generated by friction favored a better material mixing as the material was properly softened. Shoe heating further improved material mixing, but with less influence than the rotational speed and welding speed. 

Furthermore, the presence of severe porosity can be observed in welds PE_870_120, Figure 5a, and PEH_870_120, Figure 5c. The low heat generated during these welds promoted the formation of voids, such as pores and material discontinuities, which are concentrated in two main locations of the SZ, i.e., the center/AS and the border region of the RS. The center/AS of the SZ displays several round pores. Although these are undesired defects that must be avoided, they are not as problematic as the porosity found on the border of the RS. The pores and cavities found on the RS along the border with the HAZ represent a large material discontinuity. This material discontinuity is critical to the mechanical performance of the welds. The welds that presented this type of defect always failed through this zone. According to Rehman et al. [27] and Sheikh-Ahmad et al., these defects are signs of a lack of fusion during welding. The increase in frictional heat during welding by using higher rotational speeds and a lower welding speed promoted the removal of both types of defective regions. As a result, pores were not found in welds PE_1500_60 and PEH_1500_60, Figure 4b,d, respectively. Shoe heating also influenced the level of porosity, as the size and number of defects decreased in weld PEH_870_120, Figure 4c, if compared to weld PE_870_120, Figure 4a. However, the influence of rotational speed and welding speed on porosity is clearly greater. 

**Figure 6:**
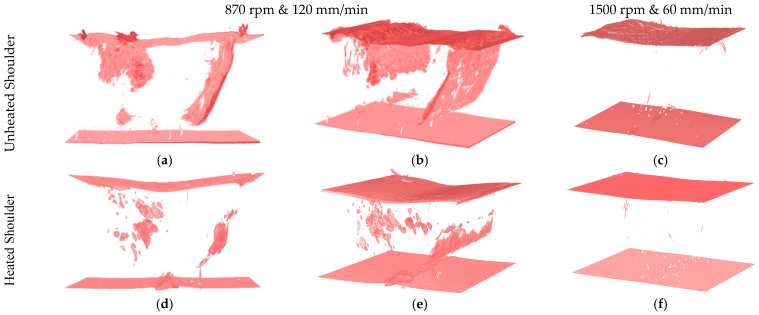
Tomography images of samples of welds (**a**) and (**b**) PE_870_120, (**c**) PE_1500_60, (**d**) and (**e**) PEH_870_120 and (**f**) PEH_1500_60.

Tomography images obtained from the samples of welds PE_870_120, PE_1500_60, PEH_870_120 and PEH_1500_60 are shown in Figure 6. 

The tomography images were obtained to better characterize the size and distribution of the voids along the joints. The images show the top and bottom surfaces of the weld samples and the internal defects. These results are in accordance with the micrographs of Figure 5 and corroborate the previous conclusions. The porosity in weld PE_870_120 is distributed along the sample, as shown in Figure 6a,b. Thus, the material discontinuity in the RS forms a wall of pores, a defect that substantially reduces the joint strength. Shoe heating contributed to the decrease in porosity, as can be understood from Figure 6d,e of weld PEH_870_120. By increasing the rotational speed and decreasing the welding speed, the porosity was completely removed, both in welds with and without shoe heating. As a result, welds PE_1500_60 and PEH_1500_60, Figure 6c,f, respectively, do not show any visible porosity on the volumes that were analyzed. 

### 3.2. Vickers Microhardness 

**Figure 7:**
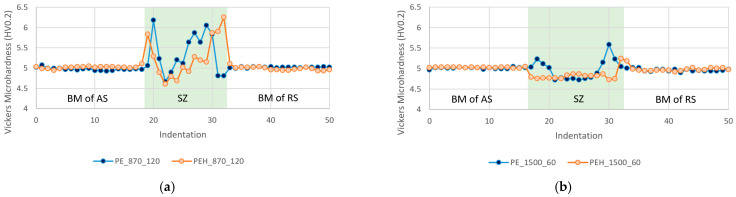
Hardness profiles of welds (**a**) PE_870_120 and PEH_870_120, and (**b**) PE_1500_60 and PEH_1500_60.

Vickers microhardness profiles were obtained from all the welds. A total of 51 indentations were evaluated on each weld, spaced by 0.5 mm, allowing the measurement of the hardness of the different welding zones. The hardness profiles of welds PE_870_120, PEH_870_120, PE_1500_60 and PEH_1500_60 are shown in Figure 7. 

The hardness of the BM of all welds on the AS and the RS coincided with the hardness of the unwelded PE specimens. Then, a short increase in hardness was generally observed on the indentations closer to the SZ, indicating that the material of the HAZ is slightly harder than the BM. This phenomenon was also observed in the study of Moreno-Moreno et al. [38].

In most welds, the border regions of the SZ on both sides revealed sharp increases in hardness, resulting in the formation of two peaks in the hardness profiles. The peak of hardness on the RS was typically higher than the peak on the AS. By increasing the rotational speed and decreasing the welding speed, the height of these peaks decreased. By using the heated shoulder, the height of the border peaks decreased even further.

The center of the SZ was always the softest region, being even softer than the BM. For welds produced with lower rotational speeds and higher welding speeds, the reduction in hardness was greater. Thereby, welds produced with higher rotational speeds and lower welding speeds had SZs of hardness closer to the BM harness. The decrease in hardness on the SZ was also previously reported by Vijendra and Sharma [29], Moreno-Moreno et al. [38] and Romero et al. [30]. The hardness variation could be associated with the variation of the crystalline content. According to Gao et al. [44] and Moreno-Moreno et al. [38], the crystalline content of the SZ is typically less than in the parent material due to physical changes in the polymer structure. 

In summary, the hardness variation was significantly reduced by increasing the rotational speed and reducing the welding speed. Shoe heating further contributed to the decrease in the hardness variation, but only in a residual way.

### 3.3. Ultimate Tensile Strength

**Figure 8:**
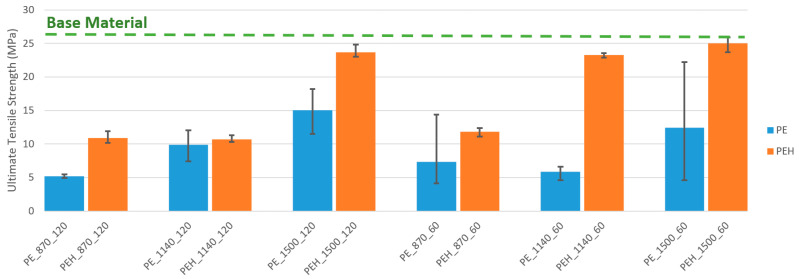
Graphical representation of average, minimum and maximum ultimate tensile strengths of each welding condition.

Tensile strength tests clearly demonstrate the influence of rotational speed, welding speed and shoe temperature parameters on joint strength. Figure 8 displays the graphical representation of average, minimum and maximum ultimate tensile strengths for each welding condition. 

Generally, for the same welding speed and shoe temperature, the increase in rotational speed led to the increase in joint strength. Only weld PE_1140_60 did not respect this trend, as the ultimate tensile strength of weld PE_870_60 was greater than the ultimate tensile strength of PE_1140_60. However, weld PE_1140_60 was produced with a plunge depth of 5.5 mm, and the others with a plunge depth of 5.7 mm. As mentioned in the morphological analysis, the reduction in penetration depth led to the formation of severe flash defect, an increase in internal porosity and a significantly larger root defect. As a result, the benefit of using a higher rotational speed was insufficient to overcome the drawback of a reduction in plunge depth. This means that Figure 8 indirectly shows that joint strength is also significantly influenced by plunge depth, and that a 5.7 mm of plunge depth is preferable compared to a penetration of 5.5 mm. For the same welding speed and shoe temperature, the maximum ultimate tensile strength was always achieved with the maximum rotational speed of 1500 rpm. 

Regarding welding speed, for the same rotational speed and shoe temperature, joint strength was normally higher with 60 mm/min than with 120 mm/min. Still, two exceptions were found. The ultimate tensile strength of weld PE_1140_120 was higher than the ultimate tensile strength of weld PE_1140_60. This exception can again be explained by the use of a shorter plunge depth on weld PE_1140_60. The other exception was weld PE_1500_120, which had a greater ultimate tensile strength than weld PE_1500_60. In this case, the loss of joint strength should be associated with the increase in surface defects. The increase in heat generated by friction resulted in more burned and degraded material at the crown surface of weld PE_1500_60. The loss of joint strength due to material degradation was also reported by Rehman et al. [27]. As mentioned in the morphological analysis, welds produced with heated shoe do not present material degradation or any other type of surface defect. 

Then, for the same rotational speed and welding speed, the ultimate tensile strength of welds produced with the shoe heated at 85 ± 5 °C was always higher than the ultimate tensile strength of welds produced with unheated shoe. Shoe heating promoted the formation of defect free surfaces and the decrease in internal porosity, which contributed to the production of stronger welds. 

In addition, the process with heated shoe was remarkably more consistent, since there was greater proximity between the maximum and minimum ultimate tensile strengths of different specimens under the same welding conditions. The dispersion was reduced, ensuring greater repeatability. On the other hand, welds produced with unheated shoe showed an unpredictable dispersion that tended to be quite large. This phenomenon can be explained, for example, as the distribution and size of the porosities, as well as the formation of surface defects not being homogeneous along the welds produced with the unheated tool. 

The weld with the best mechanical performance was PEH_1500_60, followed by welds PEH_1500_120 and PEH_1140_60, with an average ultimate tensile strength of about 25, 23.7 and 23.3 MPa, respectively. In terms of joint efficiency, these welds presented efficiencies of 97, 92 and 90%, respectively. Within welds produced without heated shoe, the best mechanical performance was achieved by PE_1500_120, which showed an average ultimate tensile strength of about 15 MPa, representing a joint efficiency of only 48%. The current work is not the first demonstrating the feasibility of FSW of PE with joint efficiencies above 90%. However, most of the publications found achieved these results with lower welding speeds. Mostafapour and Azarsa [34], Azarsa et al. [12] and Azarsa and Mostafapour [33] reported that joint efficiencies of about 95% were achieved with an optimum welding speed of 25 mm/min. Azarsa and Mostafapour [33] attempted to weld with a welding speed of 100 mm/min and obtained a joint efficiency of about 80%. With a welding speed of 70 mm/min, Eslami et al. [2,21] managed to produce welds with joint efficiencies of 97%. In our experiments, a maximum joint efficiency of 97% was obtained with a welding speed of 60 mm/min. On the other hand, a weld with a joint efficiency average of 92% was produced at 120 mm/min, which based on the literature found is the highest welding speed reported that achieved joint efficiencies above 90%. It is important that the FSW process can be carried out progressively with higher welding speeds so that it can become an increasingly competitive process at industrial level [33]. It is important to highlight the fact that these joint efficiencies were achieved with a single passage of the tool.

### 3.4. Elongation at Break

Although the tensile strength results show that the base material strength of PE is achievable by FSW, a massive drop in elongation at break was constantly observed on welded joints. The PE base material exhibits a ductile fracture with progressive stable necking and an elongation at break greater than 850%. On the other hand, all the welded joints showed brittle fracture when compared to the parent material. Furthermore, all specimens failed outside of the weld nugget, in the interface between the SZ and HAZ of the RS. The literature review confirms that the drop in elongation at break is a typical observation on PE joints by FSW, as well as brittle fracture on the RS [2,12,27,28,34]. In the current work, the elongation at break was generally greater for higher rotational speeds, lower welding speed and heated shoe. Similar observations were reported by Rehman et al. [27] and Sheikh-Ahmad [28], who mentioned that the higher process temperatures promote a better heat diffusion throughout the thickness and a better molecular mobility, resulting in greater elongations at break. 

**Figure 9:**
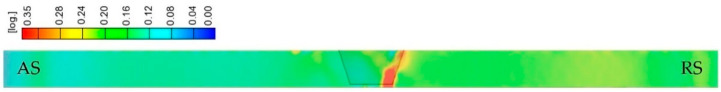
Local strain fields acquired by DIC of weld PEH_1500_60.

The maximum elongations at break were achieved in the specimens of weld PEH_1500_60, with an elongation at break of about 28%. Figure 9 shows the local strain fields acquired by DIC immediately before mechanical failure in a tensile strength test of a PEH_1500_60 weld specimen. The local strain field shows that there was deformation in the entire specimen. However, the deformation in the RS of the SZ, in the border with the HAZ, is more concentrated. Consequently, the specimen was fractured in this zone. Similar results and conclusions were obtained for the other welds. 

### 3.5. Fracture Mode Analysis

**Figure 10:**
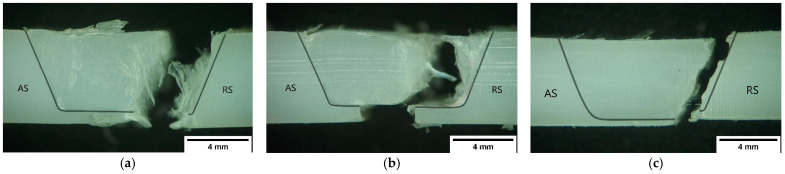
Examples of the (**a**) first fracture mode (PE_870_120), (**b**) second fracture mode (PE_1140_60) and (**c**) third fracture mode (PEH_1500_60).

Three main fracture modes were observed in the current study. Regardless of the fracture mode observed, all specimens failed in the RS of the SZ, in the border region with the HAZ, as illustrated in Figure 10. The fracture modes are related to the defects found in the weld seam that were previously discussed during the morphological analysis.

The first fracture mode, shown in Figure 10a, was found on welds produced with lower rotational speeds and a higher welding speed, such as welds PE_870_120 and PEH_870_120. The material discontinuity formed along the border of the SZ on the RS due to the accumulation of pores and weakened the joint in this area. Consequently, the fracture propagated along this region. As a result, irregular fracture surfaces with the presence of characteristic filament structures were formed.

Although all welds presented root defects, generally, the fracture occurred out of this region, except in the welds that failed with the second fracture mode. The second fracture mode is represented in Figure 10b and was found on welds produced with large root defects. The mechanism of fracture of the second mode is similar to the mechanism of the first mode, as the welds produced with short plunge depths contain material discontinuities as well. Therefore, the fracture propagated along the porosities on the border of the SZ of the RS as in the first mode. However, there are some differences between the first and second fracture modes. In the first, the fracture keeps the direction of fracture propagation, breaking out of the root defect. In the second mode, the fracture travels around the edge of the SZ until it reaches the center of the base of the weld seam, ending with the separation of the plates through the root defect, as in this region the plates are not joined. Irregular fracture surfaces with similar filament structures were found in this fracture mode.

Finally, the third mode is shown in Figure 10c. The third mode was found on welds produced with a higher rotational speed and lower welding speed, such as in welds PE_1500_60 and PEH_1500_60. Although these welds presented defect-free weld seams without any visible voids, the fracture locations are the same as those observed in the first fracture mode. The rupture also occurred along the border of the SZ of the RS, which was a region where a great variation of hardness was measured. 

The border of the SZ on the RS is the region where the material flow is more turbulent, promoting the formation of material discontinuities. Furthermore, this region consistently shows a strong change in hardness that could be associated with a variation of crystalline content, which, according to Saeedy and Givi [24], affects the tensile strength. For all these reasons, this region was always the break zone in all fracture modes.

As the first and second fracture modes were characteristic of defective welds, they generally occurred in weaker joints. On the other hand, the joints with a higher ultimate tensile strength always broke with the third fracture mode.

## 4. Conclusions

In this study, polyethylene plates were successfully butt-joined by friction stir welding using a non-conventional stationary shoulder tool. The main conclusions that can be drawn are:

1. Welds produced with unheated shoe presented burned and irregular weld surfaces. Shoe heating significantly improves weld surface finish, as it promotes the formation of defect free surfaces.
2. The increase in rotational speed, decrease in welding speed and the use of heated shoe improve joint efficiency, as these conditions favor the improvement of the material flow, the growth of SZ, the reduction in the root defect and the elimination of porosity.
3. Although all joints failed with fragile behavior in the border region of the SZ of the RS, three main fracture modes were identified. A correlation exists between fracture modes and joint efficiency. The best weld properties are achieved when the third fracture mode occurs.
4. Defect-free welds with an average joint efficiency of up to 97% were obtained. Among these, a weld with an average joint efficiency of 92% was produced at 120 mm/min, which based on the literature found is the highest welding speed reported that achieved a joint efficiency greater than 90%.

## Figures and Tables

**Table 1:** Main properties of PE.

Ultimate tensile strength	25.8 MPa
Elongation at break	>850%
Glass transition temperature [22,38]	−120 °C
Melting temperature [22,38]	130 °C
Vickers microhardness	5 HV

**Table 2:** Process parameters and their levels.

Parameter	Level 1	Level 2	Level 3
Rotational speed (rpm)	870	1140	1500
Welding speed (mm/min)	60	120	-
Plunge depth (mm)	5.5	5.7	-
Shoulder temperature	Not heated	Heated at 85 °C	-

**Table 3:** Protocol of the welding tests.

Designation *	Plunge Depth (mm)	Rotational Speed (rpm)	Welding Speed (mm/min)	Shoulder Heating Temperature (°C)
PE_870_60	5.7	870	60	Not heated
PE_1140_60	5.5	1140	60	Not heated
PE_1500_60	5.7	1500	60	Not heated
PE_870_120	5.7	870	120	Not heated
PE_1140_120	5.7	1140	120	Not heated
PE_1500_120	5.7	1500	120	Not heated
PEH_870_60	5.7	870	60	85 ± 5
PEH_1140_60	5.7	1140	60	85 ± 5
PEH_1500_60	5.7	1500	60	85 ± 5
PEH_870_120	5.7	870	120	85 ± 5
PEH_1140_120	5.7	1140	120	85 ± 5
PEH_1500_120	5.7	1500	120	85 ± 5

* The prefix PEH or PE designates whether the weld was produced with or without shoe heating, respectively, and is followed by the rotational speed and welding speeds used on each case.

## Data Availability

Not applicable.
